# Late‐Life Aerobic Exercise Attenuates DNA Damage and Telomere Dysfunction in Non‐Atheroprone but Not in Atheroprone Aortic Regions

**DOI:** 10.1111/acel.70196

**Published:** 2025-08-27

**Authors:** Jisok Lim, John Kim, Hossein Abdeahad, Shelby A. Hall, Lisa A. Lesniewski, Anthony J. Donato

**Affiliations:** ^1^ Department of Internal Medicine University of Utah Salt Lake City Utah USA; ^2^ Department of Nutrition and Integrative Physiology University of Utah Salt Lake City Utah USA; ^3^ Geriatric Research, Education, and Clinical Center, Salt Lake City Veterans Affairs Medical Center Salt Lake City Utah USA; ^4^ Nora Eccles Harrison Cardiovascular Research and Training Institute, University of Utah Salt Lake City Utah USA; ^5^ Department of Biochemistry University of Utah Salt Lake City Utah USA

## Abstract

Cellular senescence is a state of persistent cell cycle arrest and is a critical contributor to arterial aging. The primary drivers of cellular senescence are the DNA damage response (DDR) and telomere dysfunction, which is induced by increasing exposure to DNA‐damaging stimuli such as atheroprone shear stress. While late‐life aerobic exercise is an effective intervention to mitigate arterial aging, its specific impact on the DDR and telomere dysfunction is unknown and may not show uniform benefits across aortic regions subjected to atheroprone and non‐atheroprone shear stress. This study investigates the influence of late‐life aerobic exercise on DDR and telomere dysfunction in endothelial cells (EC) and vascular smooth muscle cells (VSMC) within the aortic regions exposed to distinct shear stress patterns. Old male C57BL6 mice were randomly assigned to a negative control (NC) group and habitual voluntary wheel running (VWR) groups for 16 weeks. The habitual VWR groups were further categorized into low (LR), moderate (MR), and high running (HR) groups based on their daily running distance throughout the intervention. EC and VSMC DDR and telomere dysfunction in NC, LR, and MR groups were comparable across the aortic regions. Interestingly, EC DDR and telomere dysfunction were mitigated in the non‐atheroprone aortic regions in HR, but not in VSMC. These improvements were independent of telomere length. Collectively, these data provide evidence that late‐life aerobic exercise selectively mitigates DDR and telomere dysfunction in ECs within non‐atheroprone aortic regions, rather than atheroprone aortic regions, in an exercise volume‐dependent manner, independent of telomere length.

## Graphical abstract

After 16 weeks of habitual voluntary wheel running, the aged aorta showed reduced genomic instability in non‐atheroprone aortic regions, such as the major aortic arch and thoracic aorta, but not in atheroprone aortic regions, such as the minor aortic arch, as evidenced by decreased DNA damage and telomere dysfunction.

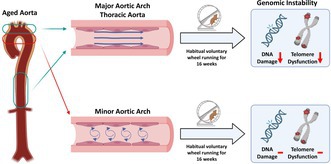

1

Aging is the primary risk factor for the development of cardiovascular diseases (CVD), a leading cause of death in the United States (National Center for Health 2017). Advancing age is associated with arterial dysfunction preceding the onset of age‐related CVD, thereby serving as a reliable predictor for future CVD morbidity and mortality (Lakatta and Levy 2003). As arterial dysfunction advances with aging, the burden of cellular senescence increases across multiple vascular beds, including the aorta, kidneys, and brain (Cohen et al. 2021; Kiss et al. 2020; Yokoi et al. 2006). This observation suggests that cellular senescence contributes to age‐related pathological processes in arterial dysfunction.

Cellular senescence is a state of persistent cell cycle arrest. A primary inducer of cellular senescence is DNA damage and telomere dysfunction (He and Sharpless 2017; Schumacher et al. 2021). Telomeres, comprised of the repeated DNA sequence (TTAGGG) located at chromosome ends, play a critical role in maintaining genomic stability (de Lange 2009). Telomere shortening occurs as cells undergo a finite number of replications. When telomere shortening surpasses a critical threshold, it triggers DNA damage response (DDR) and telomere dysfunction, ultimately leading to cellular senescence (He and Sharpless 2017; Schumacher et al. 2021). However, as advanced age is often accompanied by chronic disease states, cells are increasingly exposed to DNA‐damaging stimuli such as disturbed shear stress, reactive oxygen species, and pro‐inflammatory cytokines, which accelerate stress‐induced premature senescence (SIPS) (Bloom et al. 2023).

Shear stress is a frictional force exerted by the blood flow and has significant physiological consequences on arteries, particularly in endothelial cells (ECs) and vascular smooth muscle cells (VSMCs). Due to the morphological differences across arterial regions, such as the aorta, distinct shear stress patterns are generated. Atheroprone regions, such as the minor aortic arch, experience irregular and non‐uniform disturbed shear stress (DSS), while non‐atheroprone regions, such as the major aortic arch and thoracic aorta, are subjected to linear and uniform laminar shear stress (LSS). Previously, several studies demonstrated that ECs subjected to DSS exhibit greater SIPS compared to those exposed to LSS, which induces a lower SIPS response (Akimoto et al. 2000; Warboys et al. 2014). In addition, co‐culturing ECs with VSMCs leads to similar cellular responses to these divergent shear stress patterns in both cell types, partly mediated through intercellular signaling (Akimoto et al. 2000). This effect may be driven by the higher metabolic cost associated with cell rearrangement under DSS, which increases mitochondrial reactive oxygen species production (Yamamoto et al. 2023). We also confirmed that there is greater DDR and telomere dysfunction in the atheroprone region compared to the non‐atheroprone regions of the aorta with advancing age (Bloom et al. 2022). Nevertheless, the impact of interventions that modify shear stress, such as aerobic exercise, on DNA damage and telomere dysfunction remains unexplored.

Aerobic exercise is a lifestyle intervention recognized for its protective effect on arterial aging (Seals et al. 2008). The positive impact of aerobic exercise on arterial health can be attributed, in part, to the improvement of shear stress profiles characterized by increased LSS and reduced DSS (Suh et al. 2011). While the adoption of aerobic exercise in later stages of life is still effective in mitigating arterial aging phenotype (Gioscia‐Ryan et al. 2021), the association between late‐life aerobic exercise and DNA damage, as well as telomere dysfunction, remains unclear. Additionally, the impact of late‐life aerobic exercise, particularly in regions exposed to atheroprone and non‐atheroprone shear stress, may not yield uniform benefits, necessitating further investigation.

To address these gaps, the present study aimed to examine the extent of DNA damage and telomere dysfunction in ECs and VSMCs across atheroprone and non‐atheroprone aortic regions in old mice, including the major aortic arch, minor aortic arch, and thoracic aorta. Furthermore, we sought to explore whether engaging in late‐life aerobic exercise had an impact on DNA damage and telomere dysfunction in ECs and VSMCs within atheroprone and non‐atheroprone aortic regions.

We utilized 21 male C57BL6 mice, aged 18–19 months, which were single‐housed and provided with normal chow and water ad libitum. Mice were randomly allocated into negative control (NC, *N* = 6) group and habitual voluntary wheel running (VWR; Columbus Instruments, Columbus, Ohio) groups (*N* = 15). All groups were given access to the running wheel for 16 weeks. However, the running wheel in the NC group was locked to prevent rotation. The habitual VWR groups were then categorized into tertiles based on their daily running distance over the duration of the intervention, resulting in low (LR), moderate (MR), and high (HR) habitual running groups (*N* = 5 for each group). The average age post‐intervention at the endpoint was 22.52 ± 0.14 months. The VWR distance/day was significantly greater in HR compared to LR and MR groups (75.9 ± 11.2 vs. 21.3 ± 1.6 and 5.8 ± 2.0 m/day, respectively; *p* ≤ 0.0002; Figure 1A). To assess the impact of VWR within each group, we conducted a maximal treadmill test to determine the aerobic capacity (374.3 ± 53.9, 313.7 ± 17.6, and 251.6 ± 29.2 m; HR, MR, and LR, respectively; *p* = 0.102). We observed a significant correlation between VWR distance/day and maximal treadmill running distance (R^2^ = 0.354, *p* = 0.019, Figure 1B), and maximal treadmill running time (*R* ^2^ = 0.332, *p* = 0.025). It is important to note that direct comparisons of daily running distances across studies may be limited due to differences in wheel apparatus specifications (e.g., resistance, radius) and longer interventions (e.g., early peak in activity followed by a gradual decline), particularly in old mice.

**FIGURE 1:**
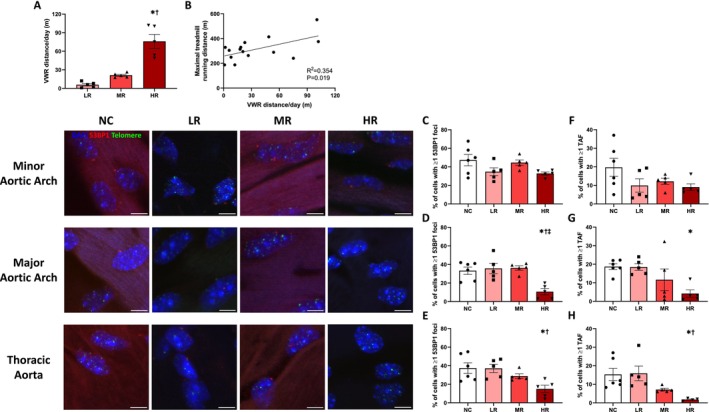
Effect of late‐life aerobic exercise on DNA damage and telomere dysfunction in endothelial cells (ECs) across atheroprone and non‐atheroprone aortic regions of the aorta. (A) Categorization of late‐life aerobic exercise groups. *N* = 5–6/group. (B) Correlation between maximal treadmill running distance and habitual voluntary wheel running (VWR) per day. *N* = 15. (C–E) Percentage of ECs with one or more 53BP1 foci in minor aortic arch, major aortic arch, and thoracic aorta, respectively. *N* = 5–6/group. (F–H) Percentage of ECs with one or more telomere‐associated DNA damage foci (TAF) in minor aortic arch, major aortic arch, and thoracic aorta, respectively. *N* = 4–6/group. LR, low habitual running; MR, moderate habitual running; HR, high habitual running; NC, negative control. Data are mean ± SEM. *vs. NC, *p* ≤ 0.039. †vs. LR, *p* ≤ 0.033. ‡vs. MR, *p* = 0.002. Scale bar is 10 μm.

Large conduit arteries accumulate DNA damage and telomere dysfunction with advancing age (Bloom et al. 2022). Although aerobic exercise in later life has been shown to alleviate arterial dysfunction, the extent to which it contributes to reducing DNA damage and telomere dysfunction remains uncertain. In addition, some regions of the aorta are in default exposed to atheroprone and non‐atheroprone shear stress throughout the lifespan. As atheroprone aortic regions preferentially accumulate DNA damage and telomere dysfunction (Bloom et al. 2022), aerobic exercise may not provide uniform benefits in aortic regions experiencing different shear stress patterns. Here, we performed enface immunofluorescence‐fluorescent in situ hybridization (IF‐FISH) to assess the impact of late‐life aerobic exercise on DDR and telomere dysfunction in both atheroprone and non‐atheroprone regions of the aorta. DDR and telomere dysfunction were evaluated using 53BP1 foci and telomere‐associated DNA damage foci (TAF), respectively. These indicators were examined in ECs and VSMCs from aortic regions experiencing contrasting shear stress patterns such as the minor aortic arch, exposed to DSS, and the major aortic arch and thoracic aorta, exposed to LSS.

The percentage of ECs with one or more 53BP1 foci remained consistent across all VWR groups in minor aortic arch (*p* = 0.077, Figure 1C). In contrast, HR showed a lower percentage of ECs with one or more 53BP1 foci compared to NC, LR, and MR in the major aortic arch, and compared to NC and LR in the thoracic aorta (*p* ≤ 0.004 and *p* ≤ 0.039, Figure 1D,E, respectively). To evaluate whether DDR was mitigated by a greater volume of aerobic exercise, we determined the correlation between the percentage of ECs with one or more 53BP1 foci and VWR distance/day. Interestingly, we found a significant correlation between the major aortic arch and thoracic aorta but not in the minor aortic arch (Figure 1SA–C). Similarly, the percentage of ECs with one or more TAF was reduced in HR compared to NC in the major aortic arch and NC and LR in the thoracic aorta but not in the minor aortic arch (*p* ≤ 0.039 and *p* = 0.372, respectively; Figure 1F–H). A significant correlation was observed in the thoracic aorta but not in the major and minor aortic arch (Figure S1D–F).

The percentage of VSMCs with one or more 53BP1 foci and TAF was not changed across all groups regardless of the aortic region (*p* ≥ 0.212, Figure 2A–F). In addition, telomere length was not different across all groups regardless of the aortic regions (*p* ≥ 0.359, Figure S2) in both ECs and VSMCs. Taken together, these results suggest that DNA damage and telomere dysfunction were alleviated by a greater volume of aerobic exercise in ECs, but not in VSMCs. Furthermore, this improvement was specific to non‐atheroprone regions.

**FIGURE 2:**
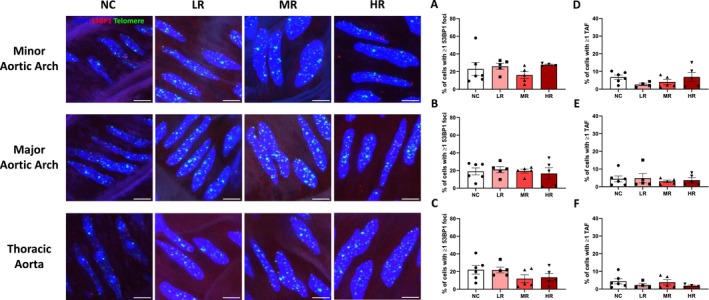
Effect of late‐life aerobic exercise on DNA damage and telomere dysfunction in vascular smooth muscle cells (VSMCs) across atheroprone and non‐atheroprone regions of the aorta. (A–C) Percentage of VSMCs with one or more 53BP1 foci in minor aortic arch, major aortic arch, and thoracic aorta, respectively. *N* = 4–6/group. (D–F) Percentage of VSMCs with one or more telomere‐associated DNA damage foci (TAF) in minor aortic arch, major aortic arch, and thoracic aorta, respectively. *N* = 4–6/group. NC, negative control; LR, low habitual running; MR, moderate habitual running; HR, high habitual running. Data are mean ± SEM. Scale bar is 10 μm.

Shear stress is a crucial factor influencing DDR and telomere dysfunction, which ultimately leads to cellular senescence. Partial ligation, which induces DSS, in the carotid artery accelerates EC senescence compared to the contralateral side without partial ligation (Nam et al. 2009). In addition, the minor aortic arch had greater senescence‐positive ECs compared to the major aortic arch in high‐fat fed mice (Warboys et al. 2014). Indeed, our previous findings showed that with advancing age, DNA damage and telomere dysfunction were more pronounced in ECs compared to VSMCs in atheroprone regions of the aorta, as opposed to non‐atheroprone regions (Bloom et al. 2022). Interestingly, the current study indicates a potential association between late‐life aerobic exercise and reduced DNA damage and telomere dysfunction in old ECs. These improvements were exclusively present in non‐atheroprone aortic regions after a certain threshold of exercise volume was reached. Although the mechanistic interpretation of this observation may need further investigation, long‐lasting exposure to detrimental DNA‐damaging stimuli such as DSS in certain aortic regions may not be responsive to late‐life aerobic exercise and thus may require preventative intervention earlier in life.

Interactions between ECs and VSMCs are essential for maintaining proper vessel wall function. Both cell types have been shown to respond to shear stress in a unidirectional manner, highlighting the crosstalk between ECs and VSMCs. The induction of LSS not only reduces the rate of proliferation in ECs but also in VSMCs (Akimoto et al. 2000). Additionally, exposure of ECs to LSS inhibited the atherogenic phenotypic shift of VSMCs compared to static conditions through endothelial signaling (Zhou et al. 2013). These results align with previous observations that both ECs and VSMCs are prone to DNA damage with aging in vivo (Bloom et al. 2022). In our study, aerobic exercise impacted ECs but not VSMCs, suggesting that such interventions may not necessarily reverse the aging processes in both cell types and may require different DNA damage repair mechanisms.

Telomere length gradually decreases with aging in humans and mice, but it remains unclear whether aerobic exercise initiated later in life can restore telomere length. While some studies suggest that aerobic exercise can improve telomere length (LaRocca et al. 2010), others have shown that DNA damage foci in senescent cells, induced by external DNA‐damaging agents, are often located at telomeres, even when telomeres are long (Hewitt et al. 2012). This indicates that persistent telomere‐associated damage can result from genotoxic stress and contribute to SIPS, independent of telomere length. Our findings align with this, showing that telomere length was comparable across all groups and aortic regions (Figure S2), while aerobic exercise reversed aging‐induced DNA damage and telomere dysfunction in ECs at the non‐atheroprone aortic region.

The current study provides several novel insights. First, the benefits of late‐life aerobic exercise did not provide uniform benefits to the DNA damage response and telomere function across the aorta. While ECs in LSS exposed non‐atheroprone aortic regions, that is, major aortic arch and thoracic aorta, showed improvement, ECs in DSS exposed atheroprone aortic regions, that is the minor aortic arch, were unresponsive. Second, the beneficial effects observed in ECs did not extend to VSMCs. Third, the improvements in DDR and telomere dysfunction were independent of telomere length. Lastly, the beneficial effects of late‐life aerobic exercise exhibited a clear exercise volume‐dependent improvement, with greater benefits observed in ECs only when a certain threshold of exercise volume was achieved. Therefore, these data provide evidence that late‐life aerobic exercise selectively mitigates DDR and telomere dysfunction in ECs within non‐atheroprone aortic regions in an exercise volume‐dependent manner, highlighting the region‐specific benefits of late‐life aerobic exercise on arterial aging.

## Author Contributions

Jisok Lim, Lisa A. Lesniewski, and Anthony J. Donato conceptualized and designed the study. Jisok Lim and John Kim conducted experiments. Jisok Lim, Hossein Abdeahad, and Shelby A. Hall analyzed data. Jisok Lim, Lisa A. Lesniewski, and Anthony J. Donato interpreted the results and drafted the manuscript. All authors reviewed and approved the final version of the manuscript.

## Conflicts of Interest

Anthony J. Donato serves as a scientific advisor and holds equity in Recursion Pharmaceuticals, while Lisa A. Lesniewski is an equity holder in the same company. None of the research conducted in collaboration with Recursion Pharmaceuticals is addressed or included in this manuscript. The remaining authors report no conflicts of interest.

## Supporting information


**Figure S1:** acel70196‐sup‐0001‐FigureS1.docx.

**Data S1:** acel70196‐sup‐0002‐DataS1.docx.

## Data Availability

The data supporting the findings of this study are available from the corresponding author upon reasonable request.
