# Supplementary figures and images for: Late‐Life Aerobic Exercise Attenuates DNA Damage and Telomere Dysfunction in Non‐Atheroprone but Not in Atheroprone Aortic Regions
Source: Aging Cell. 2025 Aug 27;24(10):e70196. doi: 10.1111/acel.70196 (PMC12507407; doi:10.1111/acel.70196)

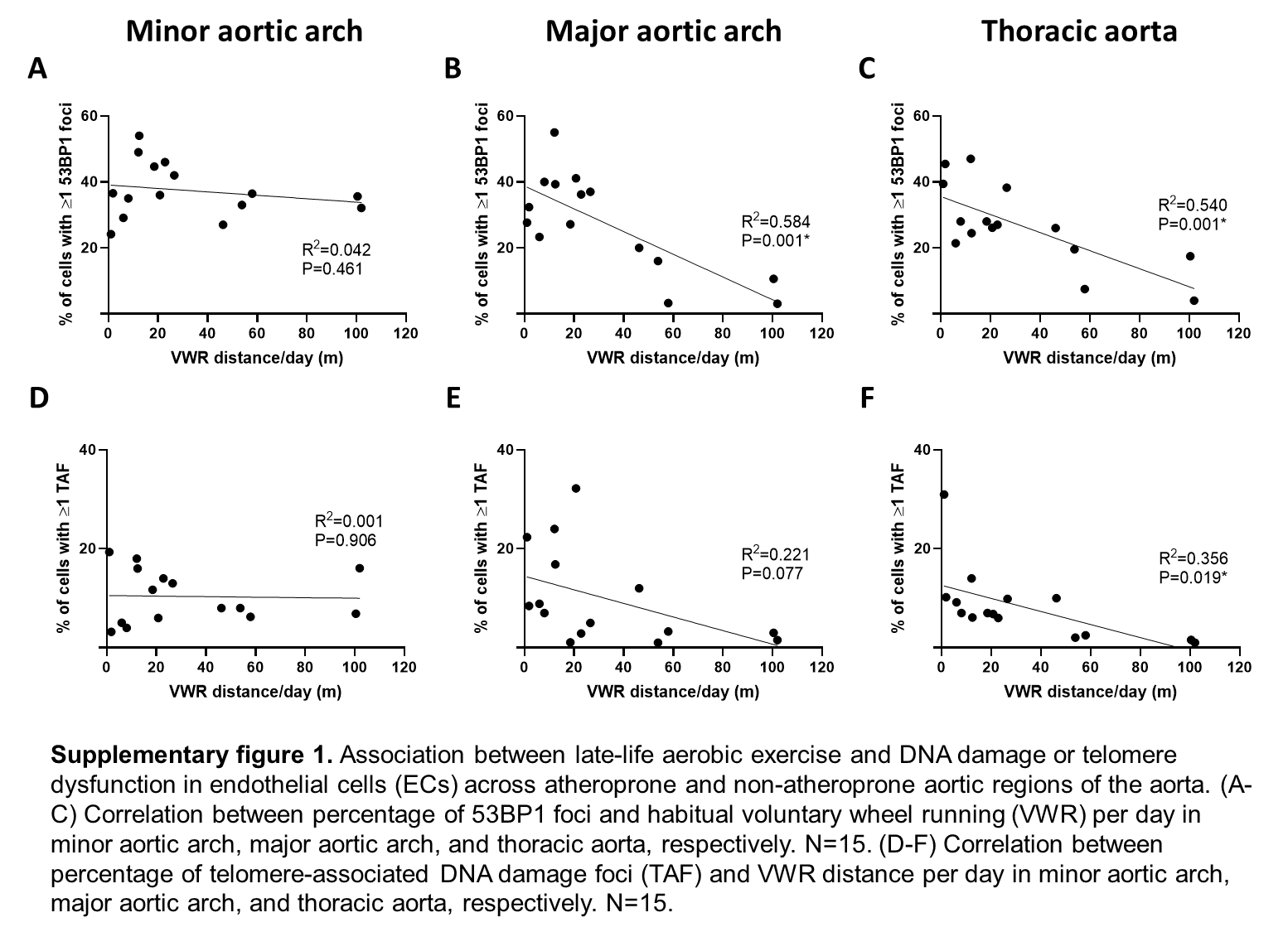


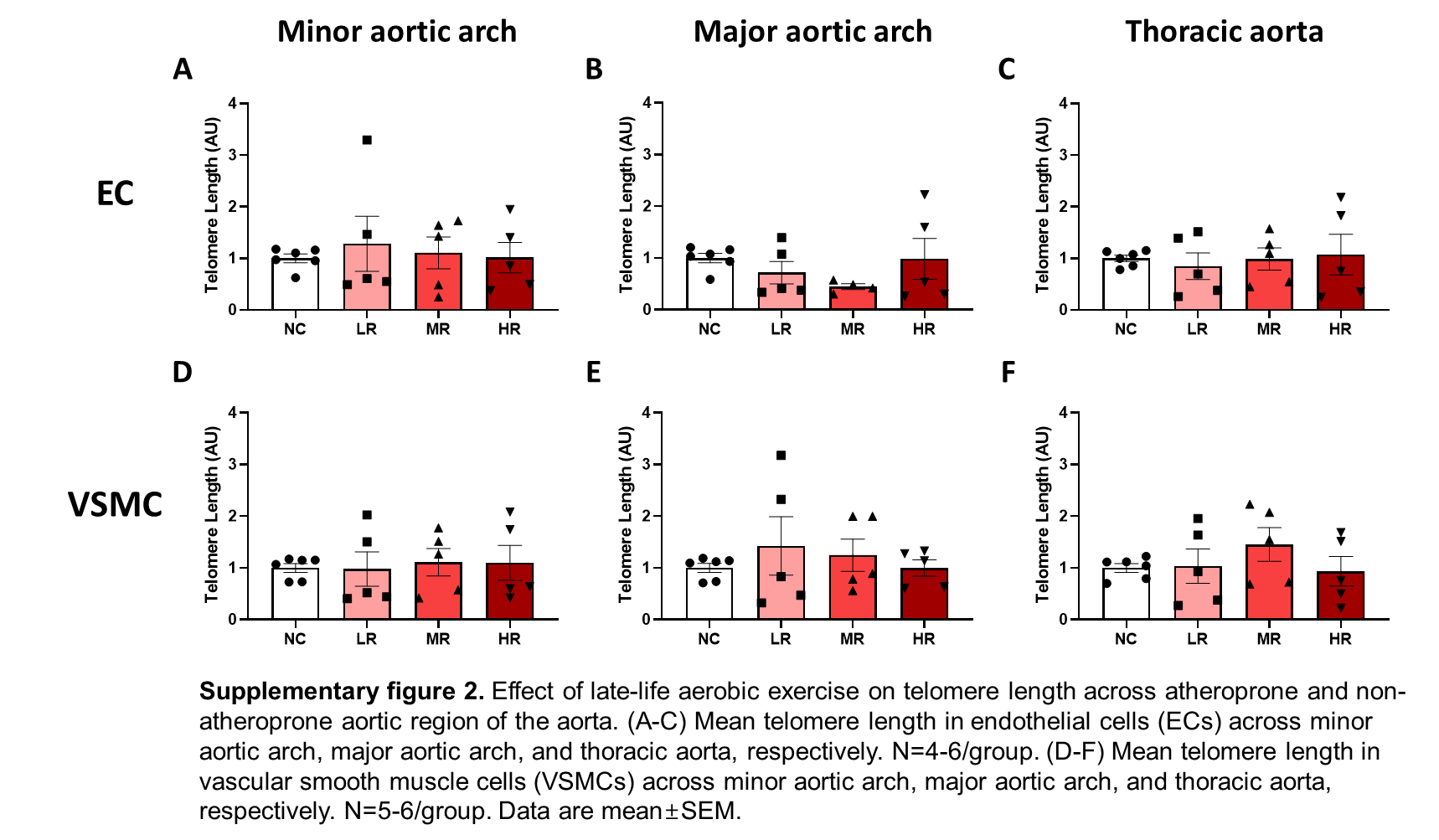

Supplement: Supplementary file 1 — Figure S1: acel70196‐sup‐0001‐FigureS1.docx. [file ACEL-24-e70196-s001.docx]
