# Supplementary material for: Late‐Life Aerobic Exercise Attenuates DNA Damage and Telomere Dysfunction in Non‐Atheroprone but Not in Atheroprone Aortic Regions
Source: Aging Cell. 2025 Aug 27;24(10):e70196. doi: 10.1111/acel.70196 (PMC12507407; doi:10.1111/acel.70196)
Supplement: Supplementary file 2 — Data S1: acel70196‐sup‐0002‐DataS1.docx. [file ACEL-24-e70196-s002.docx]

**Methods**

**Animals**

Old male C57BL/6 mice (n=21, 18 mo) were obtained from the National Institute of Aging (NIA) colony maintained by Charles River Laboratories. They were accommodated in the animal care facility at the Veterans Affairs Medical Center-Salt Lake City, adhering to a 12:12 hour light-dark cycle and fed standard rodent chow (Teklad Diet no. 8604, Envigo) ad libitum. All animal experimentation procedures were conducted in accordance with the *Guide for the Care and Use of Laboratory Animals* and were granted approval by both the University of Utah and Veteran’s Affairs Medical Center-Salt Lake City.

**Voluntary wheel running**

Mice were individually housed and provided unrestricted access to a running wheel equipped with a wheel counter interface (Columbus Instruments) for 16 weeks. The running distance was calculated based on the number of wheel rotations and is presented as the average distance covered per 24 hours.

**Maximal treadmill running**

Mice performed maximal treadmill running exercise to assess the maximal aerobic exercise capacity. One week prior to the test, the mice underwent a three separate days of acclimatization period on a motorized rodent treadmill (Columbus Instruments). During these sessions, the treadmill speed gradually increased from 5 to 15 m/min at a 10% grade over a period of 20 minutes. Specifically, mice began running at 5 m/min and a 10% grade for 5 minutes, followed by an increase to 10 m/min for 5 minutes, and finally reaching 15 m/min for the remaining 10 minutes. A gentle tail touch with a soft brush was utilized to encourage the mice to continue running during this session. This acclimatization protocol was repeated on day 2 and 3 of the acclimatization period. In the subsequent week, the mice underwent testing for maximal aerobic exercise capacity following a standardized protocol. This involved running on the treadmill at a 10% grade for 10 minutes at 7 m/min, followed by increments in speed. Specifically, the speed was increased to 10 m/min for 5 minutes and then increased by 5 m/min every 5 minutes until reaching 30 m/min. The mice continued running until they were unable to be encouraged further by a gentle tail touch.

**En face aorta preparation**

Mice were anesthetized with 2% isoflurane, and the hepatic portal vein was surgically opened. Following this, mice underwent perfusion through the left ventricle with saline for approximately 5 minutes until the saline effluent was devoid of blood. Subsequently, mice were perfused with approximately 60 ml of 4% paraformaldehyde over a period of 5-10 minutes. The heart was carefully dissected while still connected to the aorta, and the thoracic portion of the aorta was separated from the abdominal aorta. The aortic tissue was submerged in cold physiological saline solution and pinned under a dissecting microscope. Perivascular adipose tissues and branches were carefully excised from the aorta. Subsequently, the aorta was detached from the heart at its base. The aorta was then dissected into the aortic arch and thoracic aorta segments and fixed in 4% paraformaldehyde for an additional 20 minutes. Each segment was then longitudinally opened, and the endothelial surface was positioned facing downward on imaging slides to enable direct visualization and analysis.

**Immunofluorescence-fluorescent in situ hybridization (IF-FISH)**

Following en face aorta preparation, immunofluorescence-fluorescent in situ hybridization (IF-FISH) was performed on both the aortic arch and thoracic aorta on the same day. Samples were immersed in 100% methanol at −15 °C for 15 minutes, followed by rehydration in 1×PBS for 5 minutes at room temperature. Subsequently, samples were subjected to blocking solution, comprising 1 mg/mL bovine serum albumin, 3% goat serum, 0.1% Triton X-100, 1 mM EDTA, all in 1×PBS, for 30 minutes at room temperature. Then, the samples were placed in a 1:500 dilution of 53BP1 antibody (Novus Biologicals, NB100-0304, Rabbit) in blocking solution for 1 hour at room temperature. After three washes of 5 minutes each in 1×PBS, the samples were incubated in a 1:500 dilution of Alexa Fluor 555 (Invitrogen, A-21429, Goat anti-Rabbit) in blocking solution for 1 hour at room temperature. After another three washes in 1×PBS, the samples underwent dehydration in 70%, 95%, and 100% ethanol at room temperature for 5 minutes each. Following very brief drying, the samples were immersed in a hybridization solution containing 2% Tris HCL, 60% formamide, and 5% blocking reagent derived from a 10% Roche stock, along with a 1:200 dilution of Tel Probe (Integrated DNA Technologies, 5Alex488N/CC CTA ACC CTA ACC CTA A, purification: HPLC), all in diH2O. The samples were then subjected to thermal cycling at 60°C for 10 minutes, followed by denaturation at 85 °C for 10 minutes, and hybridization at 37 °C for 2 hours. Subsequently, the samples were washed twice for 10 minutes each at 60 °C in a washing solution comprising 2×saline-sodium citrate (SSC) buffer and 0.1% Tween 20 in diH2O, followed by a brief incubation in room temperature washing solution. Finally, the samples underwent washing in 2×SSC, followed by 1×SSC, and then in diH2O, each for 10 minutes at room temperature. Under a dissecting scope, the aortic arch was laterally incised to separate the major arch from the minor arch, while thoracic aortas were longitudinally opened. The samples were placed on a coverslip with DAPI Fluoromount G (VWR-Catalog #102,092–102), endothelial side down, and gently maneuvered into the DAPI solution using forceps. A glass slide was positioned on the smooth muscle side of the artery and mounted. Samples were weighed down with a 1 kg weight for 5 minutes and stored in a lightproof container at 4 °C until imaging.

**Imaging and analysis**

Samples were visualized using an Olympus Fluoview FV1000 Confocal microscope at 100× zoom, employing consistent settings across all samples. Z-stack images with a thickness of 1 μM were captured throughout the entire nucleus. Subsequently, the images were processed utilizing CellProfiler software (https://cellprofiler.org). DNA damage was characterized by distinct 53BP1 foci, whereas telomere-associated foci (TAF) were recognized as 53BP1 foci co-localizing with telomere signals. In addition, telomere length was assessed based on fluorescence intensity. The quantification of 53BP1 foci, TAF, and telomere length was performed by an examiner blinded to the experimental groups. We analyzed 90.6 ± 1.7 and 89.8 ± 1.7 cells for ECs and VSMCs, respectively, across all aortic regions.

**Statistical analysis**

Group differences in 53BP1 foci and TAF were assessed via repeated measures one-way analysis of variance (ANOVA) with Tukey post hoc tests using GraphPad Prism 8.4.3 in each aortic region. Prior to statistical analysis, all datasets were assessed for outliers and normality. Outliers were identified and removed using the robust regression and outlier removal (ROUT) method with a false discovery rate of 1%. Normality was assessed using the Shapiro-Wilk test. When normality assumptions were violated, non-parametric tests (Kruskal-Wallis with Dunn’s post hoc test) were used in place of parametric tests. Pearson correlation analysis was performed to establish correlation between 53BP1 foci and TAF, and VWR distance per day in each aortic region. Significance was determined at p < 0.05. Data are presented as the mean ± SEM.
